# Genome-Wide Association Study of Genetic Variants Associated with Serum Albumin Levels in Chinese Winter Sports Athletes

**DOI:** 10.3390/biology15040350

**Published:** 2026-02-17

**Authors:** Tao Mei, Yanchun Li, Dapeng Bao, Xiaolin Yang, Zihong He

**Affiliations:** 1 China Institute of Sport and Health Science, Beijing Sport University, Beijing 100084, China; 2 Beijing Key Laboratory of Sports Performance and Skill Assessment, Beijing Sport University, Beijing 100084, China; 3 Key Laboratory for Performance Training & Recovery of General Administration of Sport, Beijing Sport University, Beijing 100084, China; 4 Biological Science Research Center, China Institute of Sport Science, Beijing 100084, China

**Keywords:** training monitoring, athlete genetics, blood biomarkers, elite athletes, single nucleotide polymorphism, individual differences

## Abstract

This study aimed to explore genetic variants associated with serum albumin (ALB) levels in Chinese winter sports athletes using genome-wide association analysis (GWAS) and to investigate potential regulatory mechanisms using bioinformatics annotation. A total of 382 Chinese winter sports athletes were recruited. ALB levels were compared between elite and non-elite athletes. GWAS was conducted using PLINK v1.9, with ALB as the phenotype and sex, age, and principal components as covariates. Associated SNPs were annotated using GTEx and SNPnexus. No significant differences were observed in ALB levels between elite and non-elite male or female athletes, and ALB levels in all groups followed a normal distribution. We identified 113 SNPs reaching a suggestive significance threshold (*p* < 1 × 10^−5^), with per-variant variance explained estimates (7.11–11.76%) reflecting model fit within this cohort. A stepwise regression model highlighted nine candidate SNPs that together explained 51.1% of ALB variance in the study sample. Functional annotation suggested that several variants show eQTL or sQTL signals in tissues relevant to ALB biology (e.g., liver and kidney), and pathway enrichment analyses implicated amino acid and hormone metabolism. Overall, these findings are hypothesis-generating; independent replication in additional and ancestry-matched cohorts (and follow-up functional studies) is required to confirm the robustness of the associations and clarify causal mechanisms.

## Simple Summary

Blood albumin is an important protein that reflects nutrition, metabolism, and recovery status in athletes and is widely used in training monitoring. However, albumin levels can vary greatly between individuals, and the reasons for these differences are not fully understood. This study investigated whether genetic differences contribute to variation in blood albumin levels in Chinese winter sports athletes. We analyzed blood samples and genetic data from athletes preparing for the Winter Olympic Games. Albumin levels showed clear individual differences but did not differ between higher- and lower-level athletes. We identified multiple genetic variants that were associated with albumin levels, with several key variants explaining a large proportion of the individual differences. Further analysis suggested that these genetic variants may influence albumin levels through organs such as the liver and kidneys and through pathways related to protein and hormone metabolism. These findings improve our understanding of why athletes respond differently to training and recovery and suggest that genetic information may help interpret blood test results more accurately. This work may contribute to more individualized training monitoring and health management strategies for athletes.

## 1. Introduction

Albumin (ALB), the most abundant protein in plasma, is primarily synthesized by the liver and plays a crucial role in maintaining colloid osmotic pressure, transporting lipophilic substances, hormones, and drugs, regulating acid-base balance, modulating immune function, and providing antioxidant defense [1]. Changes in serum ALB levels can reflect an athlete’s nutritional status, metabolic activity, fluid balance, and recovery process [2,3], making it a widely used biomarker for monitoring training adaptations. For winter sports athletes, prolonged exposure to cold temperatures and high-intensity exercise imposes greater demands on physiological stress responses and metabolic regulation [4]. Serum ALB levels serve not only as key indicators for assessing an athlete’s nutritional status, immune function, and overall physiological condition but also as important references for adjusting training loads and preventing sports-related injuries [5,6].

Due to the complexity of exercise and environmental stimuli, biochemical markers in athletes often exhibit individual variability [7,8]. With respect to distribution ranges, previous work suggests that the reference interval (RI) for serum ALB in athletes (39–50 g/L) is narrower than that of the general population (35–52 g/L) [9]. In that study, the cohort was predominantly Caucasian (95.04%) and largely comprised athletes aged 18–40 years (97.35%) [9]. In addition, a small-scale study (*n* = 37) reported that serum ALB levels in cross-country skiers (4.71 ± 0.22 g/L) were slightly lower than conventional reference ranges established for sedentary populations and exhibited notable inter-individual differences [6]. These variations are influenced by multiple factors, including training intensity, nutritional status, and recovery strategies [10]. In terms of training load, research has found a negative correlation between ALB levels and training intensity, with prolonged strenuous exercise often leading to a temporary decline in ALB concentration due to increased protein catabolism [11]. From a nutritional perspective, studies suggest that consuming 20 g of high-quality protein post-resistance training can maximize ALB synthesis [12]. Additionally, genetic variation plays a significant role in individual differences in ALB levels [13]. Given the complexity of these influencing factors, monitoring ALB levels in winter sports athletes should be highly individualized to optimize training and recovery interventions, with genetic factors being a critical aspect requiring further investigation.

Studies have shown that genetic factors influence baseline serum ALB levels by modulating its synthesis, degradation, and metabolic processes [13,14]. Among these genetic variations, single-nucleotide polymorphisms (SNPs) are considered critical determinants of ALB levels. Notably, some SNPs may act as expression quantitative trait loci (eQTLs) or splicing quantitative trait loci (sQTLs), potentially altering the expression level or splicing pattern of relevant genes and thereby contributing to inter-individual differences in biological processes involved in ALB synthesis and metabolism [15]. Previous research has catalogued 77 known ALB mutations, among which 12 lead to analbuminemia (serum ALB < 1 g/L) [16], most commonly through splice-disrupting variants or premature stop codons that truncate the ALB protein and reduce functional albumin production; these cases are typically detected clinically in individuals with markedly low albumin levels, sometimes accompanied by mild edema [16]. In a Korean GWAS of the albumin-to-globulin (A/G) ratio (discovery *n* = 4205; replication *n* = 4637; adjusted for age, sex, BMI, smoking, and alcohol intake), rs4561508 at the TNFRSF13B locus showed the strongest association (combined *p* = 7.80 × 10^−24^), and rs174548 at the FADS1 locus also reached genome-wide significance (combined *p* = 3.54 × 10^−8^) [17]. Additionally, a longitudinal East Asian cohort study reported a GWAS identifying 71 loci associated with hypoalbuminemia (≤4.0 g/dL), and highlighted variants (e.g., rs2894536 and rs10972486) that were also associated with blood pressure changes and incident hypertension, supporting the broader relevance of ALB-related genetic variation to cardiometabolic phenotypes [18]. The composition of study cohorts plays a crucial role in the identification and replication of genetic variants, as differences in ancestry and sampling context can lead to distinct genetic markers, allele-frequency distributions, and linkage disequilibrium patterns [19,20]. However, genetic studies focusing on ALB in Chinese populations remain limited, and evidence in athlete cohorts—particularly Chinese winter sports athletes assessed under standardized sampling conditions—has not been reported to date. Therefore, investigating ALB-related genetic markers in Chinese winter sports athletes may provide insights into the genetic basis of inter-individual variation in ALB regulation and inform biomarker interpretation in training monitoring and personalized training strategies.

Therefore, this study is the first to explore genetic variants associated with ALB levels in Chinese winter sports athletes using a genome-wide approach. Furthermore, it aims to analyze the potential biological functions of these variants, including integrating GTEx eQTLs and sQTLs evidence to support functional interpretation of the association signals, thereby providing a theoretical foundation for the development of genetic marker-based training monitoring methods and athlete selection strategies in winter sports.

## 2. Materials and Methods

### 2.1. Participants

A total of 492 Chinese winter sport athletes in preparation for the Beijing 2022 Olympic Winter Games were recruited for this study, with all participants providing written informed consent and no declinations recorded. Due to unavailable serum albumin (ALB) data for 110 athletes, the final analytical sample comprised 382 athletes with complete ALB measurements. Participant characteristics are summarized in Table 1. Inclusion criteria were defined as follows: (1) being a Chinese winter sport athlete engaged in formal training throughout the study period; (2) being free from acute illness at the time of sampling and without a history of major medical conditions that may interfere with serum albumin levels (e.g., clinically diagnosed hepatic or renal diseases); (3) having serum albumin (ALB) measured during a predefined recovery phase; and (4) providing a blood sample for genotyping and/or whole-genome sequencing, alongside signed written informed consent. Exclusion criteria included: (1) acute infection, fever, or other inflammatory conditions at blood sampling; (2) a confirmed diagnosis of chronic liver or kidney disease, or clinically abnormal liver/renal function requiring medical intervention; (3) recent major injury, surgery, or hospitalization that could alter biomarker status; (4) use of medications or interventions known to exert a substantial impact on albumin levels or fluid balance around the sampling period; and (5) missing key phenotypic data or failure to pass genetic quality control assessments. All participants were classified in accordance with the Chinese National Athlete Classification System formulated by the General Administration of Sport of China (i.e., second-tier, first-tier, elite, and international elite), and represented 18 distinct winter sport disciplines, including Nordic combined, snowboard halfpipe, snowboarding, snowboard big air, biathlon, short track speed skating, skeleton, alpine skiing, figure skating, freestyle skiing halfpipe, speed skating, ski jumping, bobsleigh, luge, mogul skiing, cross-country skiing, snowboard cross, and freestyle skiing big air. All study procedures were performed in compliance with the Declaration of Helsinki and approved by the Sports Science Experiment Ethics Committee.

### 2.2. Albumin Level Measurement

During the recovery phase of their regular training cycle, venous blood samples (5 mL) were collected from the athletes in a fasting state in the early morning. The recovery phase refers to a planned low-load period within the routine training cycle, during which no high-intensity training or competition sessions were scheduled, and blood sampling was conducted prior to any training on the sampling day. This sampling time point was chosen to approximate a stable physiological baseline within the athletes’ routine training cycle by minimizing acute exercise-induced fluctuations. Participants were instructed to maintain their usual fluid intake and to avoid alcohol and strenuous exercise on the day prior to sampling. After resting at room temperature for 30 min, the samples were centrifuged using a Thermo Fisher Scientific (Waltham, MA, USA) centrifuge at 3500 rpm for 10 min to separate serum from blood cells. The isolated serum was used for ALB concentration analysis, while the blood cells were preserved for subsequent DNA extraction. ALB concentration was measured using the UniCel DxI 800 Access automated chemiluminescence immunoassay analyzer (Beckman Coulter, Brea, CA, USA), following standardized operating procedures.

### 2.3. Genome-Wide Genotyping and Whole-Genome Sequencing

Genomic DNA was extracted from blood samples using a magnetic bead-based extraction kit (Tiangen Biotech, Beijing, China) according to the manufacturer’s instructions. DNA quality was assessed prior to downstream analyses based on concentration (≥50 ng/μL), purity (A260/A280 ratio between 1.8 and 2.0), and integrity evaluated by agarose gel electrophoresis. High-quality DNA samples meeting these criteria were used for both genotyping and whole-genome sequencing (WGS). For genotyping, high-quality DNA samples were processed using the Infinium Chinese Genotyping Array-24 v1.0 BeadChip (Illumina, San Diego, CA, USA) following the standard Illumina protocol. The genotyping workflow included preparation of the MSA6 amplification product plate generated during the upstream whole-genome amplification step; precipitation of the amplified DNA; resuspension of the DNA pellet; hybridization to the Multi BeadChip; washing of the BeadChip; assembly of the flow cell; single-base extension and staining of the LCG BeadChip; and final imaging of the BeadChip. Genotyping results were processed using GenomeStudio 2.0 for genotype calling and data export. For WGS, high-quality DNA samples were sequenced at a depth of 30× using the NovaSeq 6000 sequencing system (Illumina, San Diego, CA, USA), following the standard Illumina protocol. The sequencing workflow included DNA fragmentation, purification, end-repair, adapter ligation, adapter purification, fragment selection, library amplification, library quality control, and high-throughput sequencing. Raw sequencing data in FASTQ format from both genotyping and WGS were subjected to quality control, filtered to remove low-quality reads and adapter sequences, aligned to the reference genome, and processed for duplicate removal and standard variant calling to obtain high-quality genotypes for downstream analyses.

### 2.4. Quality Control, Genotype Imputation, and Data Merging

Before genotype imputation, quality control (QC) was performed separately for array-based and WGS-derived genotypes. For both datasets, SNPs with Hardy–Weinberg equilibrium (HWE) *p*-values < 1 × 10^−7^ were removed; loci and samples with a missing rate > 0.05 were excluded for genotyping data, while a missing rate threshold of > 0.1 was applied for WGS data. Genotype imputation was conducted using Eagle v2.4.1/Minimac4 v1.0.2 software with the 1000 Genomes Project (1 kGP) as the reference panel. Imputation quality was evaluated using the INFO/R^2^ metrics, with a threshold of R^2^ > 0.7 (INFO > 0.7) set for downstream analyses. After imputation, the genotyping and WGS datasets contained 9,994,690 and 17,410,399 variants, respectively.

To generate a unified genome-wide dataset, the imputed array-based genotypes and the WGS-derived genotypes were harmonized prior to merging. Harmonization included mapping both datasets to the same genome build, ensuring consistent reference/alternate allele representation, and performing strand checks and allele alignment to minimize cross-platform inconsistencies. For variants present in both datasets, WGS genotype calls were preferentially retained (WGS-first rule) as the primary genotype source for downstream analyses, whereas array-based genotypes were used to supplement variants or genotype entries not available from WGS after QC. Variants unique to each platform were retained after platform-specific QC and post-imputation QC. The two datasets were then merged, resulting in a final genome-wide dataset comprising 17,035,767 genetic variants.

### 2.5. Pre-GWAS Quality Control

Post-imputation quality control (QC) was performed using PLINK 1.9. SNPs were excluded if they met any of the following criteria: minor allele frequency (MAF) < 5%, Hardy–Weinberg equilibrium (HWE) *p*-value < 1 × 10^−7^, or genotype missing rate > 0.1. No individuals exceeded the predefined genotype missing rate threshold (> 0.1), and therefore no samples were excluded at the individual level. After QC, 5,718,671 SNPs remained for the GWAS analysis.

### 2.6. Data Analysis

Data entry, processing, and statistical analysis were conducted using Excel 2016, SPSS 19.0, and R. Descriptive statistics were presented as mean ± standard deviation (Mean ± SD). The Kolmogorov–Smirnov test was used to assess data normality. Independent-sample *t*-tests or Mann–Whitney U tests were applied to compare ALB concentrations among athletes from different categories and genotypes, with statistical significance set at *p* < 0.05.

Prior to GWAS, principal component analysis (PCA) was performed to assess population stratification. GWAS was conducted using PLINK 1.9, incorporating age, sex, sport event, and the first ten principal components as covariates. Genome-wide significance was set at *p* < 5 × 10^−8^, and suggestive significance at *p* < 1 × 10^−5^ [19]. The genomic inflation factor (λ) was calculated to assess potential residual stratification. Manhattan plots were generated using the CMplot package in R.

Although WGS enables the detection of low-frequency and rare variants, the primary GWAS in this study focused on common variants (MAF ≥ 5%) to improve statistical stability and reduce false-positive findings given the modest sample size. Rare-variant association testing typically requires alternative analytical frameworks (e.g., burden or SKAT-type tests) and larger sample sizes for adequate power; therefore, low-frequency variants were excluded from the main single-variant GWAS in the present work.

Previously reported serum albumin-associated loci were retrieved from the NHGRI–EBI GWAS Catalog (accessed on 5 February 2026). Linkage disequilibrium (LD) was evaluated in our cohort using PLINK. For each lead SNP, pairwise LD (r^2^) with surrounding variants within ±500 kb was calculated. The variance explained by individual SNPs on ALB levels was estimated using a simple linear model, while the overall contribution of selected SNPs was assessed using a multiple linear regression model. R^2^ values were computed as the coefficient of determination from linear regression models fitted in the study sample and therefore represent in-sample explained variance. To assess potential overfitting for multi-SNP models derived from feature selection, we performed 10-fold cross-validation. In each fold, stepwise selection was conducted using the training subset only, the resulting model was then applied to the held-out subset, and predictive performance was summarized as the test-set R^2^. SNP predictors were standardized within each training fold and the same transformation was applied to the corresponding test fold. Cross-validated R^2^ was reported as the mean ± SD across folds. To facilitate biological interpretation of GWAS signals, we integrated regulatory quantitative trait locus (QTL) evidence. eQTLs are genetic variants associated with differences in gene expression levels, whereas sQTLs are variants associated with differences in pre-mRNA splicing (i.e., relative transcript isoform usage). Functional annotation was performed for the lead variants (and the nine key variants selected for downstream modeling). eQTL and sQTL evidence was obtained from GTEx. Pathway enrichment analyses were conducted based on mapped genes using SNPnexus.

## 3. Results

### 3.1. Comparison and Distribution of ALB Levels in Winter Sports Athletes

**Figure 1:**
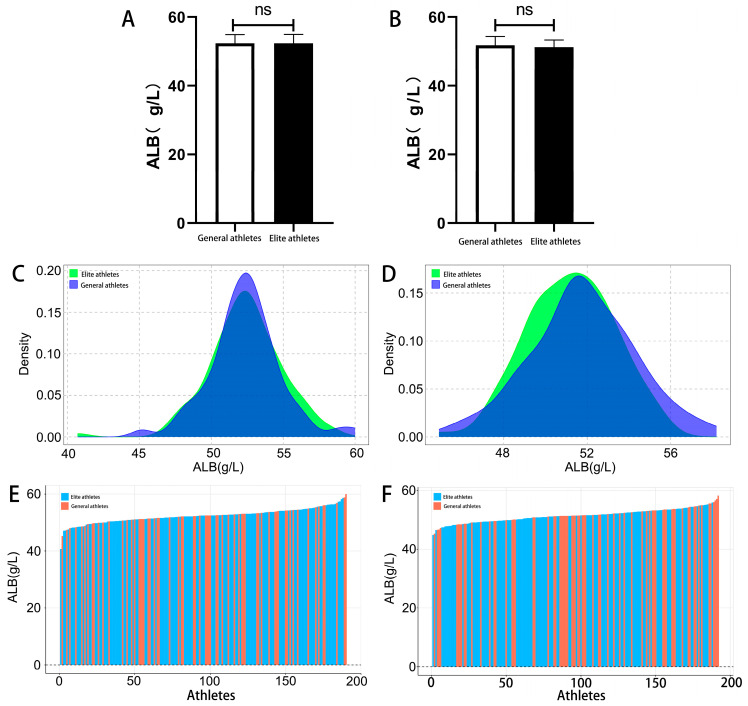
ALB concentration, distribution, and individual variability among winter sports athletes of different competitive levels. (**A**) ALB levels in male athletes; (**B**) ALB levels in female athletes; (**C**) Distribution of ALB levels in male athletes; (**D**) Distribution of ALB levels in female athletes; (**E**) Individual variability in ALB levels among male athletes; (**F**) Individual variability in ALB levels among female athletes. ns: *p* > 0.05.

In this study, athletes were categorized into two groups: sub-elite athletes (including second-class and first-class athletes) and elite athletes (including national and international masters). The results showed no significant difference in ALB levels between male elite athletes (52.36 ± 2.57 g/L) and sub-elite athletes (52.35 ± 2.50 g/L, *p* = 0.98) (Figure 1A). ALB levels in male elite athletes followed a normal distribution (*p* > 0.1), ranging from 40.70 to 58.60 g/L, while ALB levels in male sub-elite athletes also followed a normal distribution (*p* > 0.1), ranging from 45.20 to 60.00 g/L (Figure 1C), with notable inter-individual variability (Figure 1E). Similarly, no significant difference was observed in ALB levels between female elite athletes (51.22 ± 2.09 g/L) and sub-elite athletes (51.78 ± 2.53 g/L, *p* = 0.10) (Figure 1B). ALB levels in female elite athletes were normally distributed (*p* > 0.1), ranging from 44.90 to 55.90 g/L, while ALB levels in female sub-elite athletes were also approximately normally distributed (*p* = 0.08), ranging from 45.30 to 58.20 g/L (Figure 1D), with evident inter-individual variability (Figure 1F).

### 3.2. Genome-Wide Association Analysis of ALB Levels in Winter Sports Athletes

**Figure 2:**
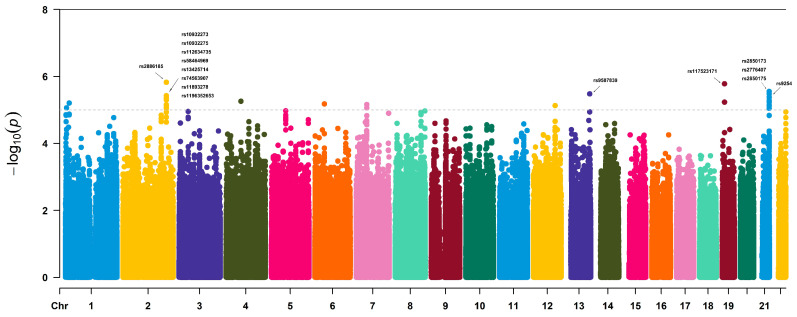
Genome-wide association analysis of ALB levels in winter sports athletes. The x-axis represents chromosomes, distinguished by different colors, while the y-axis represents −log_10_(*p*). The dashed line indicates the suggestive significance threshold (*p* < 1 × 10^−5^), and the solid line represents the genome-wide significance threshold (*p* < 5 × 10^−8^). The top 15 SNPs (as listed in Table 2) are labeled in the Manhattan plot to aid interpretation; full SNP-level details for all suggestive significant variants are provided in Supplementary Table S1.

GWAS identified 113 SNPs showing suggestive associations with ALB levels (*p* < 1 × 10^−5^; Figure 2), including rs2886185, rs117523171, and rs2850173, with a genomic inflation factor (λ) of 1.01. Based on standard gene annotations, these variants mapped to multiple genomic loci, including intragenic and intergenic regions, and were annotated to nearby or overlapping genes such as AL080251.1, ALDH4A1, TXNRD1, SPC24, COL6A1, SLC4A4, RUNX2, FIGNL1, and DDC (Table 2 lists the top 15 SNPs ranked by *p* value; the complete list of SNPs and locus annotations is provided in Supplementary Table S1). AL080251.1 locus was annotated as intronic within a non-coding intronic transcript, and detailed SNP-level information and annotations for this region are provided in Supplementary Table S1. To evaluate consistency with prior evidence, we compiled previously reported serum albumin-associated loci from the GWAS Catalog (Supplementary Table S2). There was no exact SNP-level overlap between our lead signals and previously reported lead SNP rsIDs for serum albumin. LD patterns for the suggestive SNP loci are provided in Supplementary Table S3.

### 3.3. Contribution of ALB-Associated SNPs in Winter Sports Athletes

**Figure 3:**
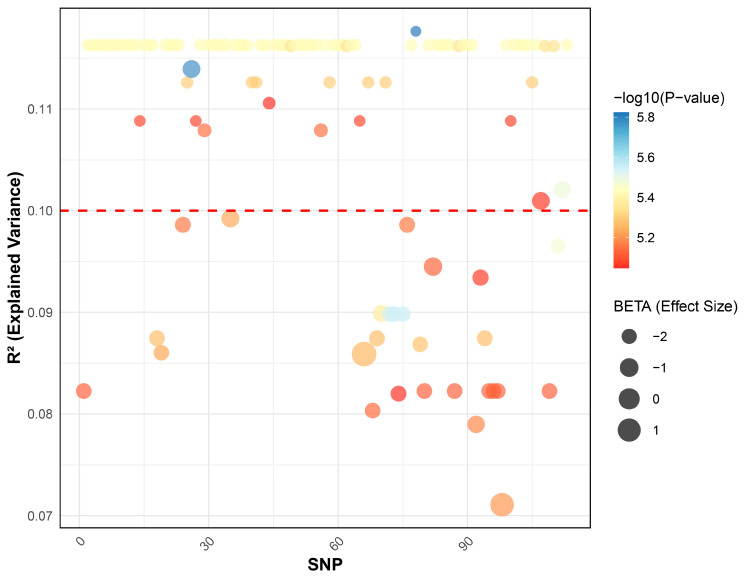
Bubble plot of effect sizes (Beta), *p*-values, and explained variance (R^2^) for ALB-associated SNPs. The x-axis represents the SNPs identified in the GWAS, while the y-axis denotes their contribution to ALB level variance. The color of the bubbles represents the *p*-value (displayed as −log_10_(*p*)), with a gradient from red to blue indicating decreasing *p*-values. The bubble size represents the estimated effect size (Beta), with larger bubbles indicating a greater effect. The red dashed line marks an explanatory power threshold of ≥10%. Detailed effect sizes for each SNP are provided in Table S1.

The percentage of variance in ALB levels explained by the 113 SNPs identified in the GWAS was calculated using both simple linear regression and multiple linear regression models (based on R^2^ values). The results showed that the variance explained by individual SNPs ranged from 7.11% to 11.76% (R^2^ = 0.0711–0.1176), while the combined explanatory power of all 113 SNPs was 54.76% (R^2^ = 0.5476) (Figure 3).

**Figure 4:**
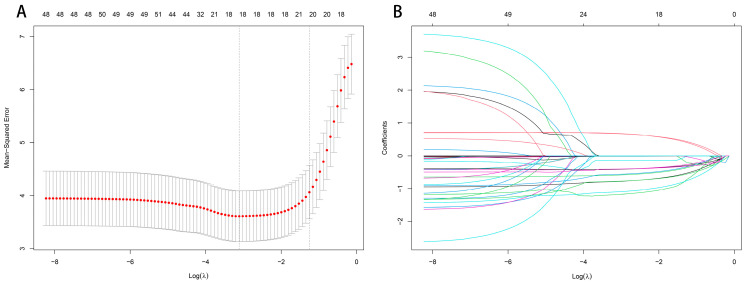
Lasso regression cross-validation curve and coefficient path plot. In panel (**A**), the x-axis represents log(λ), and the y-axis represents the mean cross-validation error. The optimal λ is selected at the point of minimum mean error. The numbers shown at the top of the panel indicate the number of predictors/SNPs with nonzero coefficients (degrees of freedom, df) at each λ. Panel (**B**) illustrates the shrinkage of regression coefficients as log(λ) changes, with nonzero coefficients at the optimal λ retained for further modeling. The numbers at the top of panel B similarly represent the number of predictors/SNPs with nonzero coefficients (df) at each λ.

Lasso regression was applied to reduce the dimensionality of the 113 SNPs, using 10-fold cross-validation to determine the optimal penalty coefficient (Lambda, λ). The optimal model was selected based on the λ value corresponding to the minimum cross-validation error, which in this study was λ = 0.04. The number of variables with nonzero regression coefficients at this λ value was recorded. Lasso regression results identified 12 SNPs (rs13383448, rs2876826, rs79061450, rs117523171, rs6941748, rs2277813, rs4077561, rs9587839, rs9254, rs114521211, rs2045007, and rs7365362) with nonzero coefficients, which were subsequently included as independent variables in the stepwise linear regression model (Figure 4).

Stepwise regression was performed using the 12 SNPs selected by Lasso regression. The results indicated that 9 SNPs (rs117523171, rs13383448, rs6941748, rs79061450, rs2876826, rs7365362, rs2277813, rs4077561, and rs2045007) had a significant impact on ALB levels in winter sports athletes. The final model yielded an R^2^ of 0.511 and an adjusted R^2^ of 0.487 (Table 3). In 10-fold cross-validation, the mean test R^2^ was 0.278 (SD 0.208; range −0.121 to 0.575).

### 3.4. Bioinformatics Analysis of ALB-Associated SNPs

To explore potential regulatory mechanisms for the nine key SNPs identified through stepwise regression, we evaluated their cis eQTL and sQTL effects using the GTEx resource.

In the eQTL analysis, rs13383448 showed significant associations in skin tissue (*p* < 0.01; intronic, non-coding). Rs2277813 exhibited eQTL signals in multiple tissues, including liver, muscle, and nervous system, with liver emphasized due to its central role in albumin synthesis and metabolism. Rs2876826 displayed significant eQTL associations in testis (*p* < 0.01), and rs7365362 showed significant eQTL signals in skin and nervous system (*p* < 0.01) (Table 4). eQTL results from other tissues are provided in Supplementary Table S4.

In the sQTL analysis, rs2277813 and rs2876826 demonstrated significant associations across kidney, muscle, and nervous system tissues, with kidney highlighted given its relevance to albumin reabsorption and urinary protein loss. Additionally, rs2876826 had significant sQTL signals in testis (*p* < 0.01) (Table 4). sQTL results from other tissues (e.g., nervous system, muscle, thyroid) are listed in Supplementary Table S4.

Most reported QTL variants are non-coding and predicted to influence gene regulation or splicing rather than protein sequence (Table 2 and Table S1). For each SNP–gene/transcript pair, we evaluated whether the variant maps within the corresponding gene region (e.g., intronic or UTR). Cohort-based LD results (Supplementary Table S3) provide additional support for locus-level interpretation where multiple variants cluster within the same LD block. These analyses collectively clarify the predicted regulatory effects of the identified SNPs and their relevance to albumin metabolism.

Signal pathway analysis of 9 ALB-associated SNPs was performed using the Reactome database. The results revealed several potential pathways linked to ALB metabolism, including amino acid metabolism, hormone metabolism, neurodevelopment, and renal disorders (Table 5).

## 4. Discussion

This study found no significant group differences in ALB levels across performance levels, although individual variations were observed. Through GWAS, we identified 113 SNPs that were suggestively associated with ALB levels in Chinese winter sports athletes. The percentage of variation in ALB levels explained by individual SNPs ranged from 7.11% to 11.76%, with the overall SNPs explaining 54.76% of the variation. The nine key SNPs included in the stepwise regression model explained 48.7% of the ALB variation (model R^2^ = 0.487). SNPs identified in this study showed eQTL or sQTL effects in tissues relevant to albumin metabolism. Notably, rs2277813 exhibited eQTL associations in the liver, while rs2277813 and rs2876826 showed sQTL associations in the kidney, highlighting their potential roles in ALB synthesis and reabsorption. Bioinformatics analysis suggested that these key SNPs may regulate ALB levels through Reactome signaling pathways, including those involved in amino acid and hormone metabolism.

In our study, the ALB levels of winter sports athletes had slightly higher lower and upper limits than the clinical reference range for adults (40–55 g/L). This pattern is broadly consistent with prior observations that ALB reference intervals in athletes may differ from the general population [9]. This observation suggests that athlete monitoring may benefit from sport- and population-appropriate reference frameworks rather than directly applying general-population reference values.

Regarding athletic performance levels, this study showed no significant differences in ALB levels between elite and non-elite winter sports athletes. This lack of difference may be explained by the relative similarity in factors such as nutrition, recovery, and training adaptations within the winter sports athlete population [21,22]. Although other studies have reported that ALB concentrations can change under extreme conditions or long-term training, such as in female cross-country skiers after long-distance competitions [23], these findings suggest that ALB is relatively stable within a well-trained and homogeneous athlete cohort. Previous studies have also reported that long-term training is associated with physiological, endocrine, and metabolic adaptations in elite athletes, which can contribute to differences in other biochemical markers (such as hemoglobin, testosterone, and urea nitrogen) compared to the general population or less trained athletes [24]. Taken together, these considerations support analyzing winter sports athletes as a single cohort for GWAS in the present study.

In this study, ALB levels in winter sports athletes exhibited individual variability. Multiple linear regression analysis showed that the 113 SNPs collectively explained 54.76% of the variation in ALB levels (R^2^ = 0.5476), suggesting that while genetic factors contribute to individual differences, 45.24% of the variation remains unexplained. This unexplained variation may be attributed to other factors not included in the model, such as training, recovery, and nutrition. Several studies have reported the impact of environmental factors on ALB levels. In a graded cycling exercise to exhaustion, ALB excretion rates increased from 5.5 ± 0.6 μg/min to 16.9 ± 2.2 μg/min during exercise [25], indicating that intense physical activity enhances ALB excretion [25]. ALB concentration also increases with exercise intensity, particularly after high-intensity exercise (85% of maximum intensity), whereas the increase is more limited following low-intensity exercise (55% or 70% of maximum intensity). These changes are closely related to physiological mechanisms such as blood volume regulation, osmotic pressure shifts, and splenic contraction [26]. Moreover, this study found that ALB concentration remains elevated post-exercise and gradually returns to baseline after 20 min of recovery [26]. Another study examining the effects of whole-body cryostimulation on exercise response and post-exercise recovery reported a significant increase in ALB concentration in cross-country skiers following exercise (from 42.2 ± 0.4 g/L to 44.6 ± 0.4 g/L) [27]. The study suggested that this increase in ALB concentration might not be due to an actual rise in ALB quantity but rather a decrease in the proportion of unbound ALB and an increase in ALB bound to lipids such as fatty acids [27]. Additionally, after eight weeks of BL-99 probiotic supplementation, cross-country skiers showed a significant decline in serum ALB levels (from 46.6 ± 1.4 g/L to 44.8 ± 0.8 g/L), suggesting that probiotic supplementation may influence ALB levels by altering metabolic pathways or gut microbiota composition [28].

Genetics is a key factor influencing ALB levels. Parent-offspring regression analysis has shown that the heritability of ALB excretion in patients with type 2 diabetes (non-insulin-dependent) is approximately 30% [29]. In the general population, the heritability of ALB excretion has been estimated at 45.2 ± 7.4% (*p* = 2 × 10^−7^), indicating a significant genetic basis for this trait [30]. These estimates support the plausibility that genetic factors contribute substantially to albumin-related phenotypes, while also indicating that environmental and physiological factors remain important contributors.

In this study, we identified 113 SNPs significantly associated with serum ALB levels in winter sports athletes, with individual SNPs explaining between 7.11% and 11.76% of the variance. Previous GWAS studies in East Asian populations (specifically in Korea) have investigated the genetic factors influencing the albumin-to-globulin ratio and identified two genome-wide significant signals (*p* < 5 × 10^−8^) along with 36 moderately significant signals (*p* < 1.0 × 10^−4^). The most notable associations were found at the TNFRSF13B locus (rs4561508, *p* = 7.80 × 10^−24^) and the FADS1 locus (rs174548, *p* = 3.54 × 10^−8^) [14]. Additionally, in a Japanese cohort, rs1260326 in the GCKR gene was reported to be associated with serum ALB levels, while rs4985726 in the TNFRSF13B gene, previously identified in the Korean cohort, was significantly associated with total protein and non-ALB protein levels in Japanese individuals [31]. Our suggestive lead variants did not show exact rsID overlap with lead variants reported in large population-based GWAS of serum albumin. Therefore, our results do not provide direct SNP-level replication of previously reported lead variants; however, they may still offer locus- or pathway-level hypotheses that warrant evaluation in independent athlete cohorts and in ancestry-matched datasets using LD proxies. This discrepancy may reflect differences in ancestry and LD patterns, genotype/imputation coverage, phenotype definition and scaling, and the specific physiological context of elite athletes assessed during a defined recovery phase. Accordingly, our findings should be considered exploratory and hypothesis-generating, and independent replication in additional athlete cohorts and/or locus-level replication using LD proxies will be required.

In our study, SNPs associated with ALB levels were primarily located in the AL080251.1, ALDH4A1, TXNRD1, SPC24, COL6A1, SLC4A4, RUNX2, FIGNL1, and DDC genes. These genes are predominantly involved in metabolic processes, antioxidation, and skeletal development. While proximity to a gene is useful for initial locus annotation, an associated SNP does not necessarily affect the closest gene. Therefore, gene assignment in this study should be viewed as a prioritization step based on convergent evidence rather than proof that the nearest gene is causal. Therefore, in interpreting the loci, we emphasized functional evidence (eQTL/sQTL) and pathway-level coherence in addition to positional mapping. While no direct evidence links all of these genes to ALB metabolism, some of them may influence ALB levels indirectly by affecting organ function. For example, in a mouse model with a Txnrd1-specific knockout, TXNRD1 deficiency activated the Nrf2 pathway through its binding to the Nqo1 and Aox1 gene promoters [32], a pathway that has been shown to stimulate ALB levels under certain conditions [33]. Previous studies have also reported that genetic variations within the ALB gene itself can directly affect ALB levels. These variations, primarily located in ALB exons, have been linked to conditions such as bisalbuminemia and analbuminemia [16].

Multiple linear regression analysis showed that the 113 identified SNPs collectively explained 54.76% of the variance in ALB levels (R^2^ = 0.5476). Stepwise regression further identified nine key SNPs, which accounted for 51.1% of the variance (model R^2^ = 0.511). Although the explained variance decreased slightly when focusing on these nine SNPs, they play a crucial role in the genetic regulation of ALB levels. Therefore, this study conducted a functional annotation of these nine SNPs to explore their potential regulatory mechanisms in ALB metabolism. eQTL and sQTL play important roles in studying gene function and disease mechanisms. At the eQTL level, our study revealed that rs2277813 exhibits eQTL effects in liver, neural, and muscle tissues, and is closely associated with the transcriptional regulation of COL6A1. Since the liver is the primary site of ALB synthesis, it plays a vital role in maintaining normal ALB levels and its physiological functions, making ALB a key biomarker of liver function [34]. The COL6A1, COL6A2, and COL6A3 genes (which encode type VI collagen) have been widely reported to be associated with liver fibrosis, where excessive collagen accumulation can lead to structural distortion and functional impairment of the liver [35]. At the sQTL level, rs2277813 and rs2876826 exhibit sQTL effects in kidney tissue, affecting the COL6A1 and DDC genes. ALB metabolism is significantly influenced by kidney function, including sodium and potassium excretion, fractional excretion of sodium and lithium, metabolic characteristics, and hemodynamics [30]. COL6A1 is involved in kidney fibrosis, pathological changes, and extracellular matrix remodeling [36], whereas DDC plays a role in the nervous system, particularly in the synthesis of dopamine and serotonin. In the kidney, dopamine is highly expressed in proximal tubules, contributing to sodium homeostasis, blood pressure regulation, and kidney disease progression [37]. Thus, DDC may regulate renal blood flow and metabolism, thereby influencing ALB excretion. Together, these eQTL and sQTL findings provide tissue-relevant regulatory clues (liver and kidney) that go beyond simple nearest-gene assumptions and help prioritize plausible locus-to-function hypotheses for follow-up. Nevertheless, the QTL evidence summarized here is derived from reference tissues and baseline conditions, and regulatory effects under training and recovery states may differ; therefore, these QTL links should be considered supportive rather than definitive.

Pathway analysis further revealed that rs2876826 in DDC is involved in catecholamine biosynthesis and amine hormone metabolism pathways, which are associated with ALB regulation. This finding is consistent with our sQTL analysis, reinforcing the regulatory role of rs2876826 in ALB levels. Additionally, this SNP may influence ALB levels by modulating serotonin and melatonin biosynthesis. Although the circadian regulation of ALB has been relatively understudied, it is closely linked to physiological processes and biomarkers such as serum calcium, phosphorus, and parathyroid hormone, all of which exhibit diurnal variations. Studies have shown that under normal conditions, serum ALB levels remain relatively stable throughout the day but exhibit significant fluctuations at night and in the early morning, typically peaking in the evening and reaching their lowest levels between 2:00 and 4:00 AM [38]. These findings provide new insights into the circadian regulation of ALB levels.

Beyond their role in hormone regulation, the nine identified SNPs are also involved in signaling pathways related to amino acid metabolism, neural development, and kidney disease. ALB consists of 585 amino acid residues, including a notable proportion of proline and glycine, which are critical for maintaining its three-dimensional structure and biological function [39]. The SNPs rs7365362 in ALDH4A1, rs2876826 in DDC, and rs4077561 in TXNRD1 are associated with metabolic pathways related to proline catabolism, glyoxylate metabolism, and glycine degradation, suggesting their potential direct involvement in ALB metabolism.

Additionally, rs2045007 in SLC4A4 has been linked to kidney disease-related pathways, particularly those associated with renal tubular acidosis caused by SLC4A4 deficiency. This finding highlights a potential connection between ALB metabolism and kidney function. SLC4A4 encodes a key bicarbonate transporter involved in acid-base balance regulation in the kidney. Since kidney disease, particularly renal tubular dysfunction, can significantly affect ALB excretion and metabolism [40], these pathways suggest that the genetic regulation of ALB levels may involve multiple physiological systems. Although the specific interactions among these pathways require further experimental validation, they provide valuable insights into the genetic mechanisms underlying ALB regulation.

## 5. Limitations

This study provides initial evidence that genetic variation may contribute to inter-individual differences in serum ALB levels in Chinese winter sports athletes, and it offers exploratory hypotheses for potential regulatory mechanisms. However, several limitations should be acknowledged. First, although we recruited a relatively large cohort for an athlete-based study, the sample size remains modest compared with large population-based GWAS, which may limit statistical power for detecting small-effect variants and may yield imprecise effect-size estimates. Second, the present work was conducted in a single cohort without an independent replication dataset; therefore, the suggestive associations and the estimated explained variance should be considered hypothesis-generating, and external replication in independent and ancestry-matched cohorts is required to confirm robustness and generalizability. Third, athletes were recruited from multiple winter sport disciplines, and although blood sampling was standardized (fasting, early morning, recovery-phase collection) and sport event was included as a covariate in the association models, residual sport-related heterogeneity and unmeasured training or hydration factors may still contribute to ALB variability. Fourth, functional interpretation primarily relied on in silico annotation (e.g., eQTL/sQTL resources and pathway enrichment), and direct experimental validation at the cellular or physiological level was not performed. In addition, pathway enrichment results are exploratory and the reported *p* values are unadjusted for multiple testing; thus, these findings should be interpreted cautiously. Finally, because multi-SNP model selection and estimation of explained variance were performed in the same cohort, the in-sample R^2^ may be inflated due to model overfitting and winner’s curse. Consistent with this concern, internal 10-fold cross-validation showed variable out-of-sample performance, underscoring the need to interpret the explained variance conservatively and to prioritize independent replication.

Future studies should include larger samples and independent replication, incorporate designs that better capture training-related dynamics and potential sport-specific effects, and perform functional experiments (e.g., cellular assays or model systems) to elucidate causal mechanisms underlying the identified loci. These efforts will strengthen inference regarding ALB regulation and clarify the potential utility of ALB-related genetic markers in athlete monitoring.

## 6. Conclusions

The SNPs rs117523171, rs13383448, and rs6941748 were identified as key genetic variants associated with ALB levels in Chinese winter sports athletes. These SNPs exhibit eQTL and sQTL effects in ALB-metabolizing organs, including the liver and kidneys, and may regulate ALB levels through amino acid metabolism, hormone metabolism, and other Reactome signaling pathways.

## Figures and Tables

**Table 1:** Demographic and ALB characteristics by sport level.

Variable	Secondary Level (M = 6, F = 7)	First Level (M = 59, F = 64)	Elite Level (M = 91, F = 87)	International Elite (M = 34, F = 34)	*p* Value
ALB_M (g/L)	52.17 ± 2.16	52.37 ± 2.55	52.51 ± 2.57	51.95 ± 2.57	0.74
Height_M (cm)	171.36 ± 6.39	175.63 ± 5.14	176.28 ± 5.09	177.26 ± 4.54	0.06
Weight_M (kg)	68.53 ± 6.89	64.39 ± 9.43	68.51 ± 8.80	70.01 ± 7.39	0.01
BMI_M (kg/m^2^)	23.40 ± 2.71	20.94 ± 3.42	22.14 ± 3.39	22.34 ± 2.78	0.07
Age_M (years)	20.50 ± 3.79	21.03 ± 3.96	21.12 ± 3.21	20.71 ± 3.54	0.93
ALB_F (g/L)	52.57 ± 0.93	51.70 ± 2.63	51.44 ± 2.07	50.66 ± 2.07	0.09
Height_F (cm)	163.91 ± 5.43	164.78 ± 5.53	165.18 ± 5.99	166.88 ± 5.91	0.33
Weight_F (kg)	57.10 ± 6.05	56.58 ± 6.94	57.48 ± 8.79	57.65 ± 7.88	0.90
BMI_F (kg/m^2^)	21.37 ± 3.24	20.87 ± 2.70	21.20 ± 3.85	20.80 ± 3.36	0.90
Age_F (years)	20.56 ± 4.92	20.76 ± 4.53	20.46 ± 4.49	20.66 ± 4.65	0.98

Note: Values are presented as mean ± SD. *p* values were obtained using one-way ANOVA across sport levels within each sex (rows with suffix _M for male and _F for female).

**Table 2:** GWAS results for ALB levels in winter sports athletes (Top 15 SNPs ranked by *p*-value).

SNP ID	CHR	Alternative Allele	GWAS_β	GWAS_P	AFR_AF	AMR_AF	EAS_AF	EUR_AF	SAS_AF	MAF	SNP-Associated Genes (Overlapped/Upstream/Downstream)	Annotation
rs10932273	2	A	−2.358	3.76 × 10^−6^	0.3192	0.0288	0.0734	0.002	0.0337	0.068	Upstream: PTH2R, Downstream: RNA5SP117	Intergenic Region
rs2886185	2	T	−2.421	1.50 × 10^−6^	0.4402	0.1873	0.2421	0.1362	0.1176	0.071	Upstream: RNA5SP117, Downstream: HSPA8P6	Intergenic Region
rs117523171	19	G	−1.312	1.66 × 10^−6^	0.8351	0.8703	0.6399	0.828	0.7526	0.408	SPC24	intronic
rs2850173	21	A	−1.915	2.81 × 10^−6^	0.9312	0.9352	0.8581	0.8777	0.7526	0.126	COL6A1	intronic
rs2776407	21	T	−1.915	2.81 × 10^−6^	0.9312	0.9352	0.8571	0.8777	0.7556	0.126	COL6A1	intronic
rs2850175	21	A	−1.915	2.81 × 10^−6^	0.9274	0.9352	0.8542	0.8777	0.7464	0.126	COL6A1	intronic
rs9587839	13	G	−1.467	3.31 × 10^−6^	0.152	0.3112	0.2827	0.3499	0.3497	0.192	Upstream: LINC01067, Downstream: LINC00399	Intergenic Region
rs9254	21	A	−1.98	3.34 × 10^−6^	0.9274	0.9366	0.8938	0.8777	0.774	0.108	COL6A1	3 utr, non-coding, 3 downstream
rs10932275	2	A	−2.358	3.76 × 10^−6^	0.2572	0.0245	0.0734	0.002	0.0307	0.068	Upstream: PTH2R, Downstream: RNA5SP117	Intergenic Region
rs112634735	2	G	−2.358	3.76 × 10^−6^	0.1467	0.0187	0.0734	0.004	0.0327	0.068	Upstream: PTH2R, Downstream: RNA5SP117	Intergenic Region
rs58464969	2	C	−2.358	3.76 × 10^−6^	0.3669	0.0389	0.0734	0.002	0.0317	0.068	Upstream: PTH2R, Downstream: RNA5SP117	Intergenic Region
rs13425714	2	G	−2.358	3.76 × 10^−6^	0.1551	0.0159	0.0734	0.002	0.0317	0.068	Upstream: RNA5SP117, Downstream: HSPA8P6	Intergenic Region
rs74563907	2	G	−2.358	3.76 × 10^−6^	0.3669	0.0389	0.0734	0.002	0.0307	0.068	Upstream: RNA5SP117, Downstream: HSPA8P6	Intergenic Region
rs11893278	2	G	−2.358	3.76 × 10^−6^	0.3669	0.0389	0.0734	0.002	0.0307	0.068	Upstream: RNA5SP117, Downstream: HSPA8P6	Intergenic Region
rs1196352653	2	T	−2.358	3.76 × 10^−6^	/	/	/	/	/	0.068	Upstream: RNA5SP117, Downstream: HSPA8P6	Intergenic Region

Note: β indicates the estimated allelic effect size on ALB levels per effect allele under the specified regression model; SE denotes the standard error of β. AFR_AF, AMR_AF, EAS_AF, EUR_AF, and SAS_AF denote the ALT allele frequencies in African, American, East Asian, European, and South Asian populations from the 1000 Genomes Project, respectively. Because allele frequencies vary across ancestries, the identity of the minor allele may differ across populations. MAF was calculated in the analyzed cohort as the frequency of the less common allele. “/” indicates that the corresponding allele frequency information was unavailable in the 1000 Genomes Project.

**Table 3:** Results of stepwise linear regression analysis.

Coefficient	Unstandardized Coefficient		Standardized Coefficient	t	*p*-Value	R^2^	Adjusted R^2^	Collinearity Diagnostics	
	B	SE	BETA					Tolerance	VIF
Constant	53.796	0.356		151.211	<0.001				
rs117523171	−0.877	0.201	−0.233	−4.363	<0.001	0.114	0.109	0.955	1.047
rs13383448	−1.472	0.391	−0.201	−3.762	<0.001	0.095	0.091	0.950	1.052
rs6941748	−0.689	0.242	−0.157	−2.847	0.005	0.075	0.073	0.897	1.115
rs79061450	−0.899	0.224	−0.215	−4.003	<0.001	0.063	0.060	0.939	1.065
rs2876826	−1.124	0.310	−0.197	−3.626	<0.001	0.044	0.042	0.919	1.088
rs7365362	0.777	0.205	0.202	3.781	<0.001	0.042	0.039	0.957	1.045
rs2277813	−0.841	0.258	−0.177	−3.264	0.001	0.038	0.037	0.920	1.087
rs4077561	−0.505	0.188	−0.145	−2.686	0.008	0.023	0.021	0.926	1.079
rs2045007	0.775	0.313	0.135	2.473	0.014	0.017	0.015	0.910	1.099

**Table 4:** eQTL and sQTL analysis of ALB-associated SNPs.

QTL	Gencode ID	Gene	SNP ID	*p*-Value	NES-Value	Tissue
eQTL	ENSG00000231908.2	IDH1-AS1	rs13383448	1.20 × 10^−4^	0.75	Skin
	ENSG00000142156.16	COL6A1	rs2277813	5.60 × 10^−6^	0.19	Liver
	ENSG00000132437.18	DDC	rs2876826	3.90 × 10^−8^	0.33	Testes
	ENSG00000159423.17	ALDH4A1	rs7365362	1.60 × 10^−5^	−0.11	Skin
	ENSG00000040487.13	SLC66A1	rs7365362	1.10 × 10^−4^	0.12	Nerve
sQTL	ENSG00000142156.16	COL6A1	rs2277813	3.80 × 10^−9^	0.86	Kidney
	ENSG00000132437.18	DDC	rs2876826	4.10 × 10^−7^	0.78	Kidney
	ENSG00000261080.1	RUNX2-AS1	rs6941748	2.10 × 10^−8^	−0.37	Testes

Note: Predicted variant consequences (e.g., intronic/UTR/intergenic) and whether the variant maps within the corresponding gene’s transcribed region are provided in Table 2 and Supplementary Table S1. All eQTL/sQTL associations reported are cis-regulatory signals from GTEx. In GTEx, the normalized effect size (NES) indicates the direction and magnitude of the allelic effect on gene expression or splicing for the tested allele (NES > 0 indicates increased expression/splicing, whereas NES < 0 indicates decreased expression/splicing).

**Table 5:** Signal Pathway Analysis of ALB-Associated SNPs.

Candidate Genes	SNP ID	Pathway ID	Description	*p* Value (Unadjusted)
ALDH4A1	rs7365362	R-HSA-70688	Proline catabolism	0.002
		R-HSA-389661	Glyoxylate metabolism and glycine degradation	0.020
ALDH4A1, DDC, TXNRD1	rs2876826, rs4077561, rs7365362	R-HSA-71291	Metabolism of amino acids and derivatives	0.001
COL6A1	rs2277813	R-HSA-419037	NCAM1 interactions	0.027
		R-HSA-186797	Signaling by PDGF	0.037
		R-HSA-2022090	Assembly of collagen fibrils and other multimeric structures	0.039
		R-HSA-375165	NCAM signaling for neurite outgrowth	0.040
		R-HSA-1442490	Collagen degradation	0.041
DDC	rs2876826	R-HSA-209905	Catecholamine biosynthesis	0.003
		R-HSA-209931	Serotonin and melatonin biosynthesis	0.003
		R-HSA-209776	Metabolism of amine-derived hormones	0.012
SLC4A4	rs2045007	R-HSA-5619054	Defective SLC4A4 causes renal tubular acidosis	0.001
		R-HSA-425381	Bicarbonate transporters	0.006
TXNRD1	rs4077561	R-HSA-2408550	Metabolism of ingested H2SeO4 and H2SeO3 into H2Se	0.003
		R-HSA-499943	Interconversion of nucleotide di- and triphosphates	0.019

Note: Pathway *p* values are unadjusted and were calculated using Fisher’s exact test based on enrichment of genes from the input gene set in each pathway.

## Data Availability

The datasets generated and analyzed in the present study are available from the corresponding author upon reasonable request.

## Supplementary Materials

The following supporting information can be downloaded at: https://www.mdpi.com/article/10.3390/biology15040350/s1, Table S1: Genome-wide association results for serum albumin (ALB) levels in Chinese winter sports athletes and annotation of associated variants; Table S2: Previously Reported Serum Albumin–Related Loci from the NHGRI–EBI GWAS Catalog; Table S3: LD Summary for Lead SNPs Associated with ALB Levels in the Athlete Cohort; Table S4: Significant GTEx eQTL and sQTL results for the queried lead variants across all GTEx tissues.
